# Management of a Rapidly Progressing Huge Pancreatic Pseudocyst

**DOI:** 10.7759/cureus.78768

**Published:** 2025-02-09

**Authors:** Brandon Weissman, Colin M Marchincin, Sumi Singh, Jeffrey J Lach

**Affiliations:** 1 Internal Medicine, Lake Erie College of Osteopathic Medicine, Buffalo, USA; 2 Internal Medicine, University at Buffalo Jacobs School of Medicine and Biomedical Sciences, Buffalo, USA; 3 Internal Medicine, University at Buffalo, Buffalo, USA; 4 Internal Medicine, Catholic Health, Buffalo, USA

**Keywords:** eus-drainage, giant pancreatic pseudocyst, pancreas, pancreas imaging, pancreatic necrosectomy

## Abstract

Pancreatic pseudocysts are a complication of both acute and chronic pancreatitis that usually develop four to six weeks from the onset of pancreatitis. Alcoholic pancreatitis is the most important risk factor for developing such cysts, although non-alcoholic cases do also occur. This is the case of a 58-year-old male patient who presented to our emergency department with a two-day history of abdominal pain, nausea, vomiting, and inability to tolerate oral food and liquids. His initial labs showed significantly elevated lipase and triglycerides. A computed tomography (CT) scan showed extensive, ill-defined pancreatic enlargement with inflammatory changes consistent with severe acute pancreatitis. Following his hospital course, on day 15, a CT abdomen/pelvis with contrast showed a new, huge pseudocyst anteriorly from the pancreas measuring 9.5 × 18 × 15 cm that rapidly progressed to 25.5 × 17.7 × 22 cm in a week with a significant mass effect. No previous sign of pseudocyst existed before this time, indicating rapid progression. Drainage and subsequent necrosectomy with stent placement were subsequently performed. To the best of our knowledge, this is the first instance of such a rapid progression of pancreatitis to large pseudocyst formation.

## Introduction

A pancreatic pseudocyst is a localized collection of pancreatic enzymes, blood, and tissue surrounded by a wall of fibrous and granulation tissue. Pseudocysts are often located outside the pancreas and usually in the lesser sac [1]. Pancreatic pseudocysts occur in approximately 0.5-1 per 100,000 adults per year [2].

Pancreatic pseudocysts often result from chronic pancreatitis but can occur from acute pancreatitis, although less frequently. Most often, these pseudocysts occur due to alcohol consumption, which accounts for 59-78% of all pseudocysts [2]. Other common causes include biliary tract diseases, blunt trauma, penetrating trauma, operative trauma, and idiopathic causes [2]. Pseudocyst pathogenesis varies by the underlying cause. In acute pancreatitis, pseudocysts are thought to result from a disruption of the pancreatic duct, causing the release of pancreatic enzymes into peripancreatic tissues. In chronic pancreatitis, two mechanisms might be involved. First, the cyst might develop from a rapid exacerbation of pancreatitis, and second, due to blockage of a branch of the pancreatic duct, which could cause a rupture of acini draining that segment of the pancreas [3]. These mechanisms allow fluid to collect and the pseudocyst to form [3].

Pancreatic pseudocysts are identified by clinical suspicion and imaging, of which a transabdominal ultrasound has a 70-90% sensitivity. The best option is a contrast-enhanced computed tomography (CT) scan of the abdomen, which carries a sensitivity of about 82-100%. Other imaging options are endoscopic retrograde cholangiopancreatography (ERCP) and magnetic resonance cholangiopancreatography (MRCP) [1]. Once diagnosed, pancreatic pseudocysts take around four to six weeks to mature fully, and spontaneous resolution occurs in approximately 33% of patients [1]. Most pancreatic pseudocysts are asymptomatic until they reach 6 cm in diameter and are considered giant once over 10 cm in diameter [4].

The standard of care involves supportive management, as pseudocysts often resolve on their own; however, this depends on the size and nature of the pseudocyst [1]. Some features, such as multiple cysts, progressive increase in size, and cysts located near the tail of the pancreas, require further intervention [1]. Interventional treatments include percutaneous drainage, which involves placing a catheter with ultrasound or CT guidance; endoscopic drainage, which can be performed via transpapillary or transmural techniques; and surgical drainage as a last resort [1]. Transpapillary endoscopic drainage involves a catheter threaded through the pancreatic duct, and stent placement connects the pseudocyst and the lumen of the pancreas. The transmural approach is performed across the stomach or duodenal wall using endoscopic ultrasound (EUS) for drainage [1].

## Case presentation

We present a 58-year-old African American male patient with a giant pancreatic pseudocyst. The patient presented to the emergency room with a two-day history of severe mid-epigastric abdominal pain and nausea. He had a past medical history of hypertension and alcohol use disorder and no surgical history. The patient had no significant family history. He had a recent history of drinking one six-pack of 12-ounce cans of beer per day.

On initial workup, he had a lipase level of 1349 U/L, aspartate aminotransferase of 232 U/L, alanine transaminase of 128 U/L, white blood cell count of 12.4 K/µL, creatinine of 5.02 mg/dL, and triglycerides of 2250 mg/dL (Table 2). The patient was hypertensive on arrival, with a blood pressure of 178/99 mmHg. The patient was promptly admitted to the intensive care unit (ICU) for medical management. On the initial non-contrast CT scan, an extensive, ill-defined, amorphous pancreatic enlargement with peripancreatic mesenteric inflammatory changes consistent with severe acute pancreatitis was seen; CT findings are organized in Table 1. The patient's pancreatitis was deemed multifactorial due to the recent alcohol use and significantly elevated triglyceride levels.

**Table 1:** Timeline of events with computed tomography scans and management CT: computed tomography; IV: intravenous; cm: centimeters; EUS: endoscopic ultrasound

Days From Admission	CT Scan Findings	Management
0	Extensive, ill-defined, amorphous pancreatic enlargement with peripancreatic mesenteric inflammatory changes, consistent with severe acute pancreatitis	Focused treatment on hypertriglyceridemia, IV fluids, and hypertension management
4	Severe edema involving the pancreas. Extensive fluid and fat stranding surrounding the pancreas. No rim-enhancing drainable fluid collection is noted	Continued with current management
15	Interval progression of pancreatitis with a huge pseudocyst extending anteriorly from the pancreas, measuring approximately 9.5 × 18.0 × 15.0 cm in size	Reached out to another facility for possible EUS drainage
19	Large mass effect upon the stomach from a very large pseudocyst, which measures approximately 20.0 x 10.4 cm, previously 18.5 x 8.9 cm at this level	Continued with current management
25	No CT scan was performed before drainage	A decision was made to drain the pseudocyst; EUS drainage occurred

**Table 2:** Laboratory values on presentation with reference ranges K/µL: thousands per microliter; g/dL: grams per deciliter; U/L: units per liter; mg/dL: milligrams per deciliter; IU/L: international units per liter; mmol/L: millimoles per liter; pH: potential of hydrogen; mmHg: millimeters of mercury; mEq/L: milliequivalents per liter; BUN: blood urea nitrogen

Parameters	Result	Reference Range
White blood cell count	12.4 K/µL	5-10 K/µL
Hemoglobin	12.7 g/dL	14-18 g/dL
Aspartate transaminase	232 U/L	10-30 U/L
Alanine transaminase	128 U/L	10-40 U/L
Total bilirubin	2.9 mg/dL	0.3-1.2 mg/dL
Alkaline phosphatase	110 IU/L	30-120 IU/L
Lipase	1349 U/L	31-186 U/L
Creatinine	4.28 mg/dL	0.6-1.2 mg/dL
Lactate	2.8 mmol/L	0.5-1.8 mmol/L
Calcium	5.2 mg/dL	4.6-5.1 mg/dL
Cholesterol	595 mg/dL	< 200 mg/dL
Triglyceride	2250 mg/dL	< 150 mg/dL
pH venous	7.26	7.35-7.45
pCO_2_ venous	53 mmHg	40-52 mmHg
HCO_3_ venous	17.9 mEq/L	21-28 mEq/L
BUN	42 mg/dL	8-23 mg/dL

On day 15, two weeks after the initial CT scan, another scan, this time with contrast, showed an interval progression of pancreatitis with a huge pseudocyst extending anteriorly from the pancreas, measuring approximately 9.5 × 18 × 15 cm in size. The collection displaced the gastric lumen anteriorly, collapsing the lumen, which contained a nasogastric tube. A large loculated fluid collection is also observed extending inferiorly. Loculated collections in both paracolic gutters are also observed. This was the first time the large pancreatic pseudocyst was seen in the patient.

**Figure 1:**
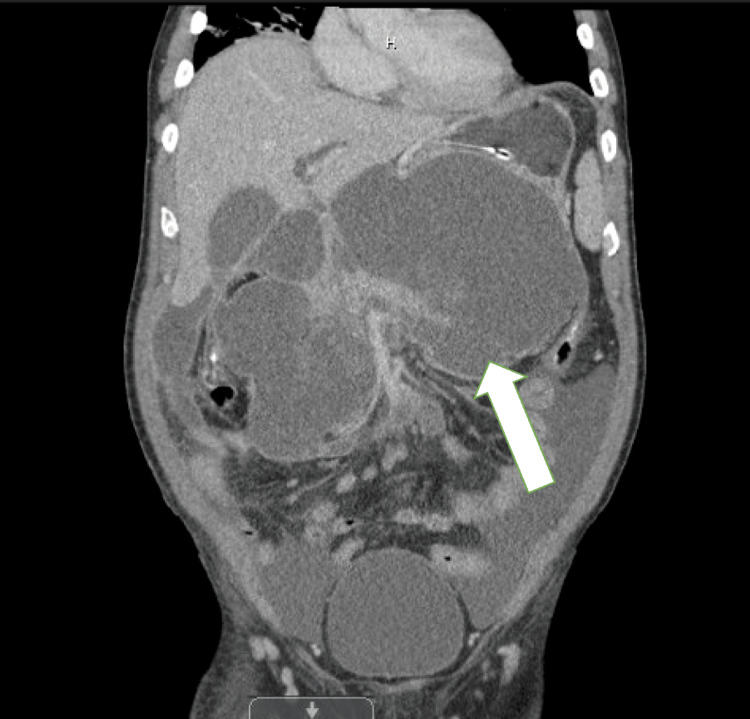
Coronal computed tomography image A large pancreatic pseudocyst (white arrow) is observed with clear delineation from the pancreas, causing a mass effect on adjacent abdominal structures.

**Figure 2:**
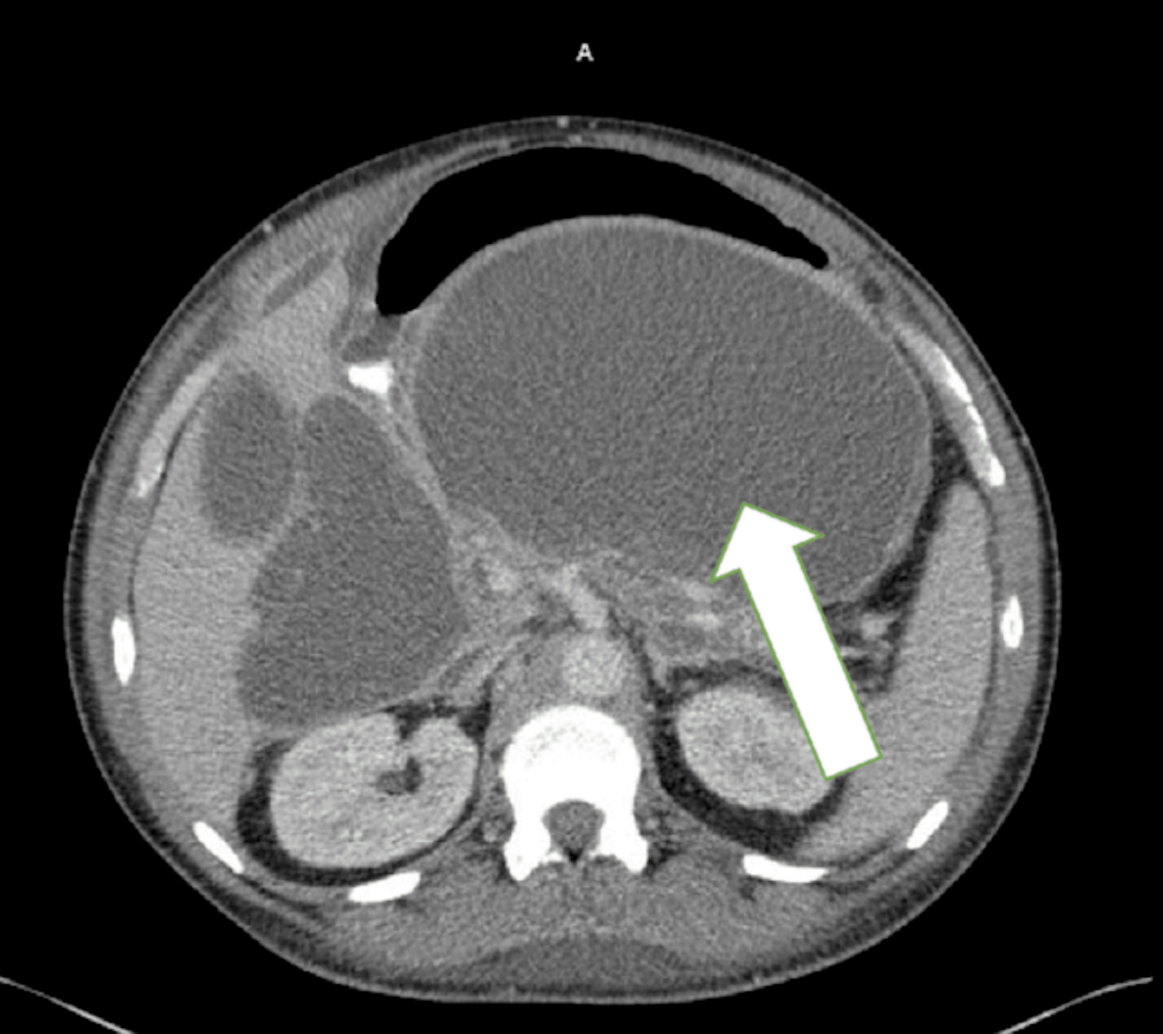
Axial computed tomography image (contrast-enhanced) A well-encapsulated cystic lesion (white arrow) is observed in the pancreatic region, compressing surrounding structures without significant internal septations.

The gastroenterology service planned to reach out to another facility for EUS necrosectomy with possible stent placement. The other facility agreed with the plan but wanted to wait a week for the pseudocyst to mature fully. The gastroenterology service wanted the pseudocyst to fully mature to allow the pseudocyst wall to become well-defined, which is associated with fewer complications. On day 19, the pseudocyst grew on CT to 20.0 × 10.4 cm and on day 21 to 25.5 × 17.7 × 22 cm, as seen in Figures 1, 2. The patient during this time had significant nausea, which was most likely attributed to the mass effect on the stomach by the pseudocyst.

**Figure 3:**
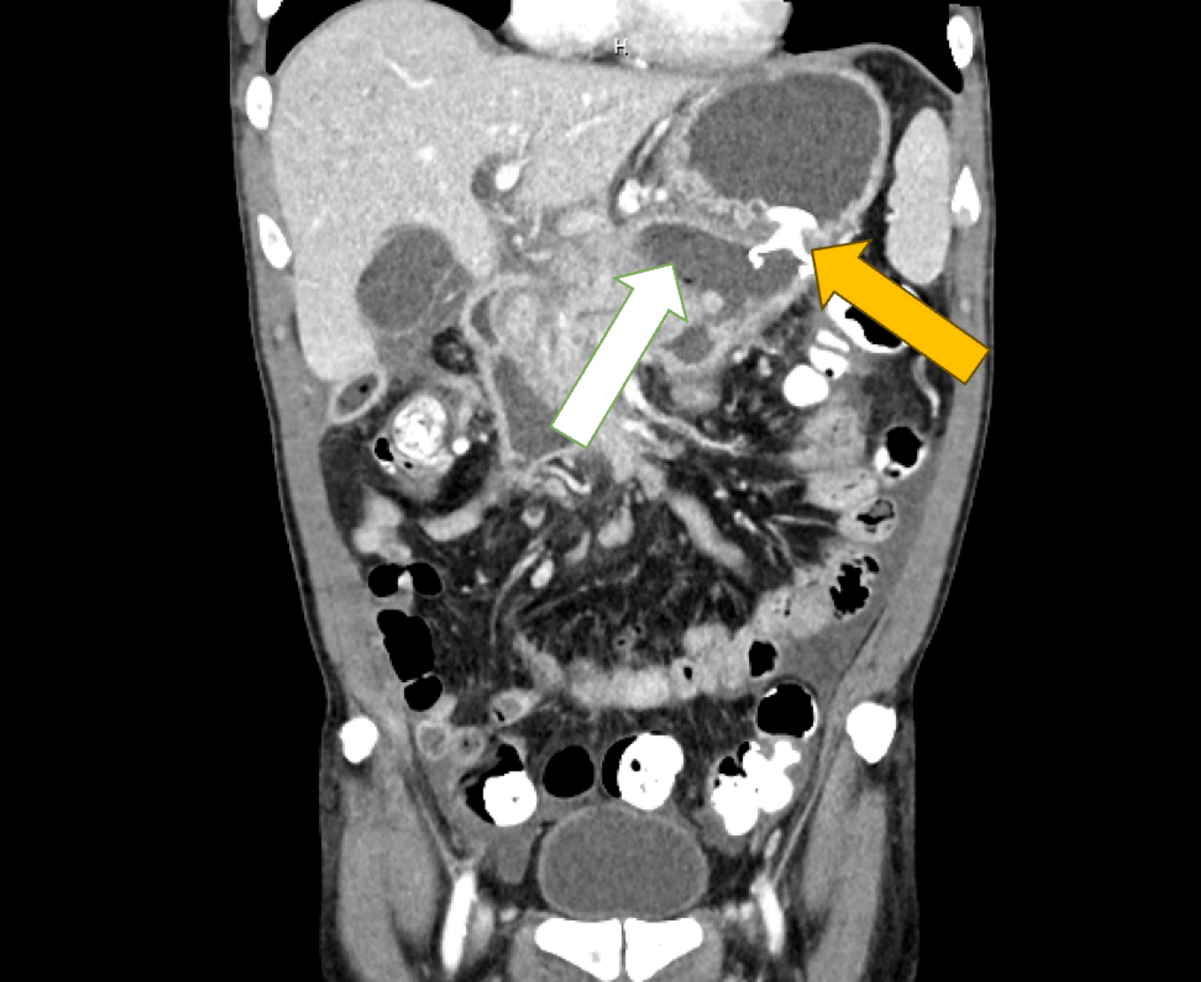
Coronal computed tomography image (contrast-enhanced) The image shows a large fluid-filled collection in the region of the pancreas, consistent with a pancreatic pseudocyst (white arrow). It has well-defined borders and adjacent bowel loops with a stent in place (yellow arrow).

EUS-guided cystogastrostomy was performed on day 25. A Boston Scientific Axios lumen-opposing metal stent (LAMS), measuring 20 mm in diameter by 10 mm in length (cautery-enhanced), was placed (Figure 3). Copious, thick, white/green fluid flowed through the stent after placement. Approximately 3.5 L of fluid was suctioned out through the scope. Balloon dilation of the stent lumen was performed under fluoroscopic guidance, progressively expanding from 12 mm to 18 mm using controlled radial expansion balloons (12 mm → 13.5 mm → 15 mm → 16.5 mm → 18 mm). The scope was advanced through the stent into the pseudocyst cavity. The pseudocyst cavity was complex, containing a moderate amount of debris. The pseudocyst cavity was irrigated, and the remaining fluid was suctioned. A small amount of the debris immediately distal to the stent was grasped with rat-tooth forceps, removed out of the pseudocyst cavity via the stent, and deposited in the stomach (necrosectomy). A total of three 10 French (Fr) double-pigtail plastic stents were then placed through the Axios stent (two 10 Fr by 5 cm long and one 10 Fr by 7 cm long).

## Discussion

Pancreatic pseudocysts are defined as a fluid collection surrounded by fibrous or inflammatory tissue without necrosis [5]. Pancreatic pseudocysts are relatively rare complications of pancreatitis that typically occur four weeks after the onset of pancreatitis. We report a case of rapid-onset pancreatic pseudocyst that grew to large proportions over a short period. The cyst first presented in the hospital on day 14 and was already considered giant in size. The patient had significant risk factors for pancreatitis, including a history of alcohol use disorder, and was found to have significantly elevated triglycerides. The patient's alcohol use preceding the pseudocyst was consistent. It is thought that severely elevated triglycerides are hydrolyzed by pancreatic lipase, causing a high concentration of free fatty acids, which will exceed the capacity of plasma albumin. This will cause free fatty acids to form micellar structures, which affect platelets, vascular endothelium, and acinar cells [6]. This causes pancreatic injury and ischemia, leading to pancreatitis development [6].

To our knowledge, this is one of the few cases in which the pseudocyst grew to over 20 cm and was the first to occur so rapidly.

Although most pancreatic pseudocysts are asymptomatic when identified, symptoms and complications can occur, especially in larger cysts, any cyst above 6 cm [4]. Complications of these large pancreatic pseudocysts mostly involve mass effects on surrounding organs. Gastric outlet obstruction, as seen in our patient, is common and presents with nausea, vomiting, and anorexia. Obstructive jaundice and even portal hypertension can also occur due to obstruction of the biliary system due to its close proximity to the pancreas [2]. Some complications with higher morbidity and mortality include splenic infarction, hemorrhage or rupture of the cyst, and even infection [5]. There was concern for peritonitis and possible sepsis in our patient, consistent with complications from pancreatitis and the pseudocyst.

The management of pseudocysts of this size has varied due to their rarity and lack of established standards for treatment. Most cysts resolve with conservative management, but those with large cysts, gastric obstruction, infection, or hemorrhage may need further intervention. Current guidelines indicate that intervention is warranted when cysts are greater than 5 cm or further complications occur. However, cyst maturation is usually preferred when considering drainage [7]. This must also be balanced with timing, as complications are more likely to occur if the cyst persists for over six weeks [8].

The literature does show some cases of these massive pseudocysts that were successfully treated; however, these patients had the cyst develop over multiple weeks compared to our patient, who presented with a large cyst and signs of obstruction and infection after two weeks. These cases all favored EUS-guided drainage due to the low level of recurrence and risks associated with open surgery. The plan to use EUS-guided drainage was then initiated on day 25 due to the severity of the complications the patient was presenting with at this point. This shows the complexity of treating these rare large pancreatic pseudocysts and the need to take into account the uniqueness of this case.

## Conclusions

We presented a case with a rapid-onset giant pancreatic pseudocyst that was diagnosed via imaging. These large pseudocysts are quite rare, and there is no defined standard of care when treating these patients, especially with such a quick onset. Initially, the treatment plan was to wait for the pseudocyst to further mature in order to better facilitate the eventual drainage. However, the onset of further complications altered our management to drain the cyst sooner. The patient began to experience symptoms of mass effect on the stomach, including nausea and vomiting, and also symptoms of peritonitis and sepsis. EUS-guided cystogastrostomy offers lower cyst recurrence rates than percutaneous drainage and similar outcomes to surgical drainage in these cases. Overall, this is an effective intervention in the treatment of giant pancreatic pseudocysts. This case, in particular, highlights the need to consider early drainage for these large cysts as the risk for complications increases.
